# Practical Hints in Pathology1Read at the meeting of the Ontario Veterinary Medical Association.

**Published:** 1902-04

**Authors:** D. King Smith

**Affiliations:** Pathologist, Ontario Veterinary College


### PRACTICAL HINTS IN PATHOLOGY.[1]

By D. King Smith, M.D.,
PATHOLOGIST, ONTARIO VETERINARY COLLEGE.

Probably the greatest difficulty one has in appearing before you is to find a suitable theme or subject that might prove both interesting and instructive. In reviewing the number of subjects that have been presented to you in past years, either before this meeting or appearing in veterinary periodicals, I was at a loss to know what to say a few words about.

Finally, I decided to wander from the usual method of writing an essay and give to you some "Practical Hints in Pathology." Before doing so I must ask the kind indulgence of those gentlemen who are acquainted with the work for taking up their valuable time.

Why this subject suggested itself to me is due to the fact that many veterinarians have not the time at their disposal to give much consideration to practical pathology, and simply by reading the description of the changes which take place during disease in various organs it is most natural for many "not to ask themselves," How is it that these changes have been studied and demonstrated?

To many, no doubt, the process has always seemed most difficult, and in my next few words I will try to show that the subject is *not* so obscure and deep, but at the present day is rather simple, due to the improvements in microscopes and other instruments.

An absolute diagnosis is often impossible to the pathologist unless he has possession of certain facts regarding the source of the specimen, its exact location, manner of growth, etc.; therefore the surgeon or practitioner should keep accurate notes regarding

[1] Read at the meeting of the Ontario Veterinary Medical Association.

every specimen that he removes and examines. This, unfortunately, is often neglected, and specimens are forwarded for microscopical examination without any history at all, and on that account seldom is a satisfactory examination made.

It is better to send more than one piece of a tissue, if possible, and some of the adjacent healthy tissue.

First, we will consider the preservation of gross specimens:

In the preparation and preservation of pathological specimens one must be guided entirely by the nature of the specimen as to the way in which it shall be prepared.

Soft specimens and organs are best preserved in fluids. Membranous parts are sometimes best inflated and dried, as dilated stomach, intestines, etc.; they should be varnished after drying.

Hollow organs like the heart may be filled with absolute alcohol or 6 per cent. formalin solution, under pressure, the openings tightly corked, and the organ immersed in a solution of the same.

After a time the heart walls become hard, and windows may be cut across, so as to expose the interior. The majority of specimens obtained will be suitable for preservation in fluids. Alcohol and formalin are the two solutions most frequently used.

*Steps.* (*a*) Wash the specimen in flowing water until the blood is removed; (*b*) put in 60 to 80 per cent. alcohol and change when the alcohol becomes cloudy.

The volume of alcohol should be much greater than that of the mass. Lately formalin in a 2 to 4 per cent. solution has come into wide use. It has the following advantages: (*a*) It is much cheaper; (*b*) preserves color—contrast is better; (*c*) does not require such frequent changes as alcohol.

The foregoing process you will find most useful in keeping any interesting specimens.

I must now say a few words as to preparing specimens for microscopical examination.

Before a specimen can be cut thin enough for examination under the microscope it must be hardened in order that the tissue be *not* injured during the process of cutting.

It is well to put the tissue into a hardening fluid as soon as possible after death. This can often be done by the person forwarding the specimen.

First, wash the organ, of which you are going to forward a piece, in flowing water until the blood is removed; then with a sharp knife cut small pieces of the organ—one-quarter to one-half inch, or thereabouts—and now place in 95 per cent. alcohol. Use alcohol in a large quantity compared to the mass. You should now seal the bottle and forward specimen.

The alcohol is changed daily for several days—five or six days—and then the specimen is ready for cutting.

Another method is to place small pieces—one-quarter by one-half inch—into a 4 per cent. formalin solution, and then in a day or so remove to 95 per cent. alcohol. By either of these methods the tissue is now ready to be cut. The cutting is done by means of instruments called microtomes, of which there are a great many varieties.

The cut sections are now kept in 95 per cent. alcohol, and may be kept indefinitely if care be used in changing the alcohol when it becomes cloudy.

If these cut sections are now examined under the microscope it would be very difficult or almost impossible to make out the structure; therefore it is necessary to resort to staining.

Certain tissues or parts of a tissue have a special affinity for staining fluids, and it is due to this fact that certain tissues stand out in relief one to the other.

The process of staining used most frequently for general work is termed "the double staining." In this method two stains are used, namely, hæmatoxylin and eosin. The steps are as follows:

1. Stain in hæmatoxylin for one and one-half to two minutes.
2. Wash in water until no more stain comes away.
3. Stain in eosin one and one-half to two minutes.
4. Wash in water until no more stain comes away.
5. Dehydration, by placing in alcohol.
6. Oil of cloves to clear specimen.
7. Mount in Canada balsam.

In this paper, gentlemen, I have not attempted to give in detail the process of staining, hardening, etc., but simply a general idea of this important branch of our work.

